# Supplementary material for: Identification and Full-Length Sequence Analysis of a Novel Recombinant Goat Astrovirus Genotype in Guangxi, China
Source: Viruses. 2024 Jul 29;16(8):1213. doi: 10.3390/v16081213 (PMC11359656; doi:10.3390/v16081213)
Supplement: Supplementary file 1 [file viruses-16-01213-s001.zip › viruses-3056036-supplementary.pdf]

Table S1. Genomic amplification primer sequence information for the goat astrovirus GX HC 2023 strain.

Name	Primer pair sequences (5′ -3′)	primer binding site (nt)	Amplified product size (bp)	Tm (℃)
1	GCGTTGATGAAGCAATTGC<br>TCCTCACTACGGATCTCGACTG	1-19<br>1087-1108	1108	58
2	GTGCGTGCTGCATCCATCA<br>ACTTGAGCATCTCCTCCACGTC	948-966<br>2187-2208	1261	60
3	TGGGTGTTTGAAACGGACACGA<br>CACCATCCAGTATCTTCTCTTTC	2097-2118<br>2941-2963	876	58
4	GCATGATAGTGAGCGGGACTAT<br>CTAGCCATTGGGAATATTCTTTGG	2812-2833<br>3872-3895	1084	58
5	GAGCAGTTGGATTATCTTTGGAG<br>AGCATGGGGTCACCCTT	3842-3864<br>4509-4525	684	57
6	GCCYAAGGAYGGATGGTACA<br>TTTCCCTTCACCTATGCAAT	4482-4500<br>6194-6213	1730	55

Table S2. Genomic amplification primer sequence information for the goat astrovirus GX WZ 2023 strain.

Name	Primer pair sequences (5′ -3′)	primer binding site (nt)	Amplified product size (bp)	Tm (℃)
1	CCAAAACAAGTCAGATTGCAATCATGTCTC<br>GGGGCCATGCTGGATCTGACCTG	1-30<br>1218-1240	1240	60
2	AGATCTGAGGATGGCAAGGTTG<br>CCATAACAATCCATGTAGTCACGCT	1131-1152<br>2856-2880	1750	59
3	GACACCCGAGTGAAGCACATAAC<br>GCCCATTGCGCACCACGAT	2771-2793<br>3966-3984	1214	60
4	GGACCATCCTTTCAAGGAGTATG<br>GTTGAGATGCAGGGCATATC	3796-3818<br>5007-5026	1231	58
5	CCTCAATTTCCGATGCTAGA<br>TTTTCCCCTTCACCTATGCTAATTAAATCAC	4984-5003<br>6254-6284	1301	55

Table S3. The nucleotide and deduced amino acid identities of the goat astrovirus GX HC 2023 strain and other astrovirus strains obtained from the Genbank.

Strain	Goat astrovirus GX HC 2023						
	Genome (%)	ORF1a (%)		ORF1b (%)		ORF2 (%)	
	nt	nt	aa	nt	aa	nt	aa
Ovine astrovirus GX	94.3	95.1	97.5	94.6	97.8	93.2	95.9
Caprine astrovirus G3.1	86.2	85.7	95.2	86.3	93.6	86.5	91.9
Caprine astrovirus G5.1	62.1	64.5	68.5	73.3	79	51.5	48.9
Goat astrovirus GX WZ 2023	62.5	65.3	67.4	72.9	78.4	52	45.1
Ovine astrovirus	42.8	40.1	25.9	57.1	49.4	34.8	22.4
Ovine astrovirus CH16	43.5	40.6	25.5	58	49.2	35.3	22
Ovine astrovirus CH17	43.8	40.5	25.2	58.5	49.4	35.9	21.9
Ovine astrovirus S5.1	62.8	65.8	68.2	72.7	79.4	53.2	49.4
Ovine astrovirus S6.1	62.3	65.2	67.6	73.2	79.2	51.2	49
Ovine astrovirus UK 2013	43.5	40.8	25.3	58.2	49.4	36	21.9
Ovine astrovirus UK 2014	43.5	40.8	25.3	58.2	49.4	36	21.9
Caprine astrovirus G2.1	55.2	59.3	56.2	65.8	67.4	45.1	35.4
Caprine astrovirus Sichuan	62	63.5	65.1	72.8	77.6	52.1	51
Caprine astrovirus SWUN F1 2019	88.6	95	98.1	95.4	97	77.7	82.9
Caprine astrovirus SWUN F2 2019	52.1	52.5	43.3	65.2	68.6	46	34.9
Human astrovirus 1 JZ	43.8	41.1	24.2	61.3	58	39.7	25.3
Porcine astrovirus 1 GX	43.7	42.3	25.8	61.4	57.8	38	25.4
Bovine astrovirus 16 2021 CHN	62.2	64.1	68.5	72.7	78.4	52.5	49.4
Bovine astrovirus EEGN4	63.1	64.9	67.3	71.8	78.2	53.7	55.8
Bovine astrovirus JPN 2015	60.7	64.9	66.9	71.7	78.4	48.8	38.7

Table S4. The nucleotide and deduced amino acid identities of the goat astrovirus GX WZ 2023 strain and other astrovirus strains obtained from the Genbank.

Strain	Goat astrovirus GX WZ 2023						
	Genome (%)	ORF1a (%)		ORF1b (%)		ORF2 (%)	
	nt	nt	aa	nt	aa	nt	aa
Goat astrovirus GX HC 2023	62.5	65.3	67.4	61.3	78.4	52	45.1
Ovine astrovirus	43.4	38.4	24.5	57.5	51.8	35.8	21.6
Ovine astrovirus CH16	43.7	39.4	25.1	57.6	50.8	36.3	22.4
Ovine astrovirus CH17	43.8	39.7	24.9	58	50.8	36	22.5
Ovine astrovirus S5.1	76.3	78.8	89.2	86.7	95	65.1	56.5
Ovine astrovirus S6.1	76	79.1	88.4	87.3	95.4	62.5	54.4
Ovine astrovirus UK 2013	43.8	38.7	25	57.5	50.8	37.4	22.5
Ovine astrovirus UK 2014	43.7	38.7	25	57.5	50.8	37.3	22.5
Caprine astrovirus G2.1	56.1	60.8	58.9	65.9	69	46.4	31.5
Caprine astrovirus G3.1	62.6	65.2	66.8	73.9	79.4	52	45.2
Caprine astrovirus G5.1	74.4	74.2	83	88.5	95.8	62.8	55.3
Ovine astrovirus GX	62.7	65.4	67.2	73.7	78.6	52.2	44.9
Caprine astrovirus Sichuan	76.8	75.3	82.5	90.6	95.4	69.8	64
Caprine astrovirus SWUN F1 2019	62.2	65.7	67.3	73.2	78.4	51.6	43.9
Caprine astrovirus SWUN F2 2019	53.6	54.3	43.8	66.2	69.8	46	32.8
Human astrovirus 1 JZ	44.5	42.2	22.8	63	59.8	38.3	25.2
Bovine astrovirus 16 2021 CHN	68.4	65.4	69.8	75.8	84.2	68.9	68.4
Bovine astrovirus EEGN4	64.9	66.7	69.9	75	84.4	55.8	42.6
Bovine astrovirus JPN 2015	63.2	64.8	68.9	75.5	84.2	51.8	37.5
Porcine astrovirus 1 GX	44.8	43.4	25.7	62.8	58.4	37	24
